# Commentary: Intraganglionic reactive oxygen species mediate inflammatory pain and hyperalgesia through TRPA1 in the rat

**DOI:** 10.3389/fpain.2024.1456548

**Published:** 2024-09-05

**Authors:** Felix Yang, Arkadeep Ghosh, Shreya Katwala, Xiang-Ping Chu

**Affiliations:** Department of Biomedical Sciences, School of Medicine, University of Missouri-Kansas City, Kansas, MO, United States

**Keywords:** TRPA1, TRPV1, ROS, pain, trigeminal ganglia

## Introduction

TRPA (transient receptor potential ankyrin 1) is the stand-alone subfamily of the TRPA family of receptors. The gene itself encodes a large protein that is expressed in humans, rodents, zebrafish, and Drosophila (1–3). Similar to the other subfamilies of TRP channels, TRPA1 is a homotetrameric, non-selective cation channel, activated by a multitude of exogenous and endogenous compounds (2–4). In the past decade, there has been a growing body of literature that have described its role in pain modulation. Several studies have shown that TRPA1 is expressed in Schwann cells, oligodendrocytes, astrocytes, primary afferent neurons, vascular endothelial cells, and other tissues that can relay nociceptive signals (5–8). It was first thought that TRPA1 served primarily in cold afferent signaling and noxious temperature sensation (9, 10). However, we now know that the channel plays a more expansive role in not only pain sensation but also chemoreception, neurogenic inflammation, and hearing (11). Other known chemomodulators of TRPA1 include cinnamaldehyde, isothiocyanate, or thiosulfinate compounds (12, 13). The significance of TRPA1 in pain research stems from recent studies demonstrating that modulation of TRPA1 has potential therapeutic benefit to the treatment of chronic pain. One recent study showed that CYP1B1-derived endogenous agonists of TRPA1 plays an important part in producing pain response (14). Deficiency of CYP1B1, an enzyme expressed mostly in mouse brain, human neurons, and astrocytes, had decreased pain-related outcomes consequently from reduced TRPA1 agonism.

While several stimuli and chemoreceptors have been established as modulators of TRPA1, one major mechanism TRPA1 transmits pain signals is sensing reactive oxidative species (ROS) with subsequent activation (15, 16). ROS plays a critical role in the development of pain of several etiologies through primarily increasing excitability in pain pathways (17, 18). Additional pathways we know ROS contribute to creating tissue inflammation, neuroinflammation, and pain include lipid peroxidation and decreased GABA release from the central nervous system (17, 19, 20). In terms of how ROS such as hydrogen peroxide, peroxyl radicals, and peroxynitrite relate to TRPA1, the recent study from Ro's laboratory describes the close relationship between ROS and TRPA1 (21). Their results ultimately reveal TRPA1 to be a promising target to directly antagonize or inhibit pain in pain medicine via reducing ROS in ganglionic neurons. To date, four TRPA1 antagonist compounds have been clinically trialed with only GRC17536 successfully passing Phase II trials. While GRC17536 showed a significant decrease in pain score within patients with diabetic polyneuropathy, issues with bioavailability and pharmacokinetics have prevented it from entering Phase III. Further, an unclear understanding of the mechanism of TRPA1 antagonist compounds and their limited effect in rodent TRPA1 limits further safety and efficacy studies (22).

## ROS-induced mechanical hyperalgesia via TRPA1

A recent study published in *Frontiers in Pain Research* from the Ro's laboratory further expanded upon the close relationship between ROS and its direct relationship with pain via TRPA1 sensitization (21). The purpose of this research was to examine whether inflammation in the masseter causes a prolonged accumulation of ROS in trigeminal ganglionic neurons. Other questions answered in this study included how ROS has concurrent effects in upregulating TRPA1 intraganglionic expression in the setting of chronic inflammation. 50% Complete Freund's Adjuvant in isotonic saline was injected to the left masseter muscles of the test group with the control group not receiving either CFA or vehicle treatment. ROS levels were measured using a cell-permeant oxidant-sensing probe 2′,7′-dichlorodihydrofluorescein diacetate (H2DCFDA) as well as a fluorescence assay. Trigeminal ganglia (TG) ipsilateral to the injection site were removed at 1, 4, 7, 14, or 28 days after CFA injection. A baseline fluorescence without H2DCFDA was measured and subtracted from resulting fluorescence which demonstrated the intensity of ROS in the treated TG.

They further assessed mechanical hyperalgesia and spontaneous muscle pain utilizing a behavioral model. Rats were trained to lean against the experimenter's hand wearing leather gloves. Von Frey Filaments were then applied to the masseter region with withdrawal of the head considered a positive response. This same model was applied to examine the role of intragangionic ROS accumulation. Rats were either given PBS, the vehicle control, or phenyl N-tert-butylnitrone (PBN), a ROS scavenger molecule. Lastly, this behavioral model was used to examine the role of TRPA1 in inflammatory mechanical hyperalgesia by administration of AP18, a TRPA1 antagonist, directly into the TG. They also tested if administration caused spontaneous pain and if AT18 attenuated this pain. They used the Rat Grimace Scale (RGS) which consisted of capturing face images of the rats by a blinded observer over the 10-minute course. Images were captured, at most, every 60 s yielding 10 images per rat.

They found that ipsilateral masseter injection with CFA resulted in ROS upregulation in the TG. This study also showed that using scavenger molecules to reduce ROS, attenuated the CFA-induced mechanical hyperalgesia, showing a correlation between ROS accumulation and mechanical hyperalgesia. AP18 was found to significantly attenuate CFA induced mechanical hyperalgesia, showing a correlation between TRPA1 and mechanical hyperalgesia. Direct ROS administration via H_2_O_2_ were shown to have a statistically significantly higher RGS score of 1.25, which was attenuated to nearly 0.5 when H_2_O_2_ and AP18 were co-administered. H_2_O_2_ treated rats were also found to have higher levels of *Trpa1* mRNA in TG compared to the control group. These results support that ROS activates TRPA1 in the trigeminal ganglion in a model of CFA induced TMJ pain. Furthermore, ROS stimulation of TRPA1 can also induce upregulation of this ion channel in the TG and this can be dampened by ROS scavenger therapies.

## Discussion

The study performed by the Ro's laboratory showed that CFA (Complete Freund's Adjuvant) induced prolonged ROS accumulation in the TG (trigeminal ganglia) leading to TRPA1 dependent hyperalgesia. This study elucidates the potential role of pharmaceuticals that either reduce ROS accumulation within somatic sensory ganglia or downregulate TRPA1 expression. For example, TRPA1 antagonist, HC-030031, was shown to reduce guarding pain behaviors after deep tissue incision even with increases in endogenous ROS and H_2_O_2_ after injection of HC-030031 (22).

Bortezomib, an antineoplastic agent, is known to induce ROS accumulation and potentially cause CIPN (chemotherapy induced peripheral neuropathy) (23). The Trevisan laboratory showed that treatment with HC-030031 or a-lipoic acid, a ROS scavenger, 7 days after administration of bortezomib in mice reversed the mechanical hypersensitivity (23). Therefore, further investigation should be done on reductions in mechanical hypersensitivity with ROS scavengers alone, TRPA1 antagonists alone, or in combination in animal models with elevated ROS accumulation due to drug administration or pathologic inflammatory conditions. Many conditions beyond temporal pain show the potential for TRPA1 antagonists to reduce hyperalgesia such as rheumatoid arthritis, endometriosis, and IBD (inflammatory bowel disease) (24). However, the use of TRPA1 antagonists and ROS scavengers has not been thoroughly explored in humans with various pathologic conditions, necessitating experiments in mouse models that can mimic these proinflammatory conditions. For example, active immunization can produce CIA (collagen induced arthritis) mouse models which should be treated with ROS scavengers, TRPA1 antagonists, and a combination of the two to evaluate changes in mechanical hyperalgesia (25).

TRPV1 (transient receptor potential vanilloid 1) is also known to be linked to hyperalgesia (26, 27). TRPV1's role with ROS is still poorly understood, however we know that both TRPV1 and TRPA1 are linked to the hyperexcitability of nociceptive afferents in the cough reflex via ROS accumulation (28). To better understand ROS accumulation effects on TRPV1 and TRPA1 in a mechanical pain-invoking condition, such as CIPN, a mouse model should be created to evaluate the differences in hyperalgesia by comparing different treatment modalities such as TRPV1 or TRPA1 antagonist alone or in combination with ROS scavengers such as a-lipoic acid.

Research has shown how ROS and TRPA1 activation in sensory terminals contribute to pain (21). That said, one important finding in Ro et al.'s paper is that ROS-TRPA1 mechanism “within sensory ganglia” is both necessary and sufficient to mediate inflammatory pain. Intraganglionic mechanisms of pain modulation occur within the sensory ganglia and contribute to the regulation and modulation of pain signals (29). They include neuropeptide release, ion channel regulation (21), immune cell interaction, glial cell activation, synaptic plasticity, and modulation of sensory neuron excitability. Understanding intraganglionic mechanisms of pain modulation is crucial for developing targeted therapies for chronic pain conditions. By targeting these mechanisms, researchers and clinicians aim to alleviate pain while minimizing side effects associated with more general pain management strategies.

## References

[1] Zheng J. Molecular mechanism of TRP channels. Compr Physiol. (2013) 3(1):221–42. 10.1002/cphy.c120001 23720286 PMC3775668

[2] Yang F Sivils A Cegielski V Singh S Chu XP. Transient receptor potential (TRP) channels in pain, neuropsychiatric disorders, and epilepsy. Int J Mol Sci. (2023) 24(5):4714. 10.3390/ijms24054714 36902145 PMC10003176

[3] Zhang M Ma Y Ye X Zhang N Pan L Wang B. TRP (Transient receptor potential) ion channel family: structures, biological functions and therapeutic interventions for diseases. Signal Transduct Target Ther. (2023) 8(1):1–38. 10.1038/s41392-023-01464-x 37402746 PMC10319900

[4] Zygmunt PM Högestätt ED. TRPA1. Handb Exp Pharmacol. (2014) 222:583–630. 10.1007/978-3-642-54215-2_23 24756722

[5] De Logu F Nassini R Materazzi S Carvalho Gonçalves M Nosi D Rossi Degl'Innocenti D Schwann cell TRPA1 mediates neuroinflammation that sustains macrophage-dependent neuropathic pain in mice. Nat Commun. (2017) 8(1):1887. 10.1038/s41467-017-01739-2 29192190 PMC5709495

[6] Hamilton NB Kolodziejczyk K Kougioumtzidou E Attwell D. Proton-gated ca(2+)-permeable TRP channels damage myelin in conditions mimicking ischaemia. Nature. (2016) 529(7587):523–7. 10.1038/nature16519 26760212 PMC4733665

[7] Story GM Peier AM Reeve AJ Eid SR Mosbacher J Hricik TR ANKTM1, A TRP-like channel expressed in nociceptive neurons, is activated by cold temperatures. Cell. (2003) 112(6):819–29. 10.1016/s0092-8674(03)00158-2 12654248

[8] Shigetomi E Tong X Kwan KY Corey DP Khakh BS. TRPA1 channels regulate astrocyte resting calcium and inhibitory synapse efficacy through GAT-3. Nat Neurosci. (2011) 15(1):70–80. 10.1038/nn.3000 22158513 PMC3282183

[9] Patapoutian A Peier AM Story GM Viswanath V. ThermoTRP channels and beyond: mechanisms of temperature sensation. Nat Rev Neurosci. (2003) 4(7):529–39. 10.1038/nrn1141 12838328

[10] Bandell M Story GM Hwang SW Viswanath V Eid SR Petrus MJ Noxious cold ion channel TRPA1 is activated by pungent compounds and bradykinin. Neuron. (2004) 41(6):849–57. 10.1016/s0896-6273(04)00150-3 15046718

[11] Guimaraes MZP Jordt SE. Chapter 11. TRPA1: a sensory channel of many talents. In: Liedtke WB Heller S, editors. TRP Ion Channel Function in Sensory Transduction and Cellular Signaling Cascades. Boca Raton, FL: CRC Press/Taylor & Francis (2007). p. 1–11. Available online at: http://www.ncbi.nlm.nih.gov/books/NBK5237/(accessed December 24, 2023).

[12] Legrand C Merlini JM de Senarclens-Bezençon C Michlig S. New natural agonists of the transient receptor potential ankyrin 1 (TRPA1) channel. Sci Rep. (2020) 10(1):11238. 10.1038/s41598-020-68013-2 32641724 PMC7343857

[13] Bautista DM Jordt SE Nikai T Tsuruda PR Read AJ Poblete J TRPA1 mediates the inflammatory actions of environmental irritants and proalgesic agents. Cell. (2006) 124(6):1269–82. 10.1016/j.cell.2006.02.023 16564016

[14] Sun L Zhang J Niu C Deering-Rice CE Hughen RW Lamb JG CYP1B1-derived epoxides modulate the TRPA1 channel in chronic pain. Acta Pharm Sin B. (2023) 13(1):68–81. 10.1016/j.apsb.2022.09.007 36815047 PMC9939319

[15] Moccia F Montagna D. Transient receptor potential ankyrin 1 (TRPA1) channel as a sensor of oxidative stress in cancer cells. Cells. (2023) 12(9):1261. 10.3390/cells12091261 37174661 PMC10177399

[16] Andersson DA Gentry C Moss S Bevan S. Transient receptor potential A1 is a sensory receptor for multiple products of oxidative stress. J Neurosci. (2008) 28(10):2485–94. 10.1523/JNEUROSCI.5369-07.2008 18322093 PMC2709206

[17] Tobore TO. Towards a comprehensive theory of non-cancer acute and chronic pain management: the critical role of reactive oxygen and nitrogen Species in pain, and opioid dependence, addiction, hyperalgesia, and tolerance. Adv Redox Res. (2021) 2:100003. 10.1016/j.arres.2021.100003

[18] Mittal M Siddiqui MR Tran K Reddy SP Malik AB. Reactive oxygen species in inflammation and tissue injury. Antioxid Redox Signal. (2014) 20(7):1126–67. 10.1089/ars.2012.5149 23991888 PMC3929010

[19] van ‘t Erve TJ Kadiiska MB London SJ Mason RP. Classifying oxidative stress by F2-isoprostane levels across human diseases: a meta-analysis. Redox Biol. (2017) 12:582–99. 10.1016/j.redox.2017.03.024 28391180 PMC5384299

[20] Yowtak J Lee KY Kim HY Wang J Kim HK Chung K Reactive oxygen species contribute to neuropathic pain by reducing spinal GABA release. Pain. (2011) 152(4):844–52. 10.1016/j.pain.2010.12.034 21296500 PMC3108328

[21] Zhang Y Asgar J Shou H Pak J Da Silva JT Ro JY. Intraganglionic reactive oxygen species mediate inflammatory pain and hyperalgesia through TRPA1 in the rat. Front Pain Res. (2023) 4:1204057. 10.3389/fpain.2023.1204057 PMC10261988 37325677

[22] Hu Z Zhang Y Yu W Li J Yao J Zhang J Transient receptor potential ankyrin 1 (TRPA1) modulators: recent update and future perspective. Eur J Med Chem. (2023) 257:115392. 10.1016/j.ejmech.2023.115392 37269667

[23] Sugiyama D Kang S Brennan TJ. Muscle reactive oxygen species (ROS) contribute to post-incisional guarding via the TRPA1 receptor. PLoS One. (2017) 12(1):e0170410. 10.1371/journal.pone.0170410 28103292 PMC5245866

[24] Trevisan G Materazzi S Fusi C Altomare A Aldini G Lodovici M Novel therapeutic strategy to prevent chemotherapy-induced persistent sensory neuropathy by TRPA1 blockade. Cancer Res. (2013) 73(10):3120–31. 10.1158/0008-5472.CAN-12-4370 23477783

[25] Landini L Souza Monteiro de Araujo D Titiz M Geppetti P Nassini R De Logu F. TRPA1 role in inflammatory disorders: what is known so far? Int J Mol Sci. (2022) 23(9):4529. 10.3390/ijms23094529 35562920 PMC9101260

[26] Caplazi P Baca M Barck K Carano RA DeVoss J Lee WP Mouse models of rheumatoid arthritis. Vet Pathol. (2015) 52(5):819–26. 10.1177/0300985815588612 26063174

[27] Bonacin YS Marques ICS Garcia SB Silva SBG Canola PA Marques JA. The role of vanilloid receptor type 1 (TRPV1) in hyperalgesia related to bovine digital dermatitis. J Dairy Sci. (2020) 103(8):7315–21. 10.3168/jds.2019-17035 32505399

[28] Westlund KN Kochukov MY Lu Y McNearney TA. Impact of central and peripheral TRPV1 and ROS levels on proinflammatory mediators and nociceptive behavior. Mol Pain. (2010) 6:46. 10.1186/1744-8069-6-46 20691059 PMC2924298

[29] Taylor-Clark TE. Role of reactive oxygen Species and TRP channels in the cough reflex. Cell Calcium. (2016) 60(3):155–62. 10.1016/j.ceca.2016.03.007 27016063 PMC5012938

